# Prediction of MAYV peptide antigens for immunodiagnostic tests by immunoinformatics and molecular dynamics simulations

**DOI:** 10.1038/s41598-019-50008-3

**Published:** 2019-09-16

**Authors:** Roger Luiz Rodrigues, Gabriela De Lima Menezes, Marielena Vogel Saivish, Vivaldo Gomes Da Costa, Maristela Pereira, Marcos Lázaro Moreli, Roosevelt Alves Da Silva

**Affiliations:** 1 Universidade Federal de Goiás, Laboratório de Virologia, Jataí, GO 75801-615 Brazil; 2 Universidade Federal de Goiás, Núcleo Colaborativo de BioSistemas, Jataí, GO 75801-615 Brazil; 3 Universidade de Brasília, Departamento de Biologia Celular, Brasília, DF 70910-900 Brazil; 4 Universidade Federal de Goiás, Laboratório de Biologia Molecular, Instituto de Ciências Biológicas, Goiânia, GO 74690-900 Brazil

**Keywords:** Infectious diseases, Molecular dynamics, Protein design

## Abstract

The Mayaro virus is endemic to South America, and the possible involvement of *Aedes* spp. mosquitoes in its transmission is a risk factor for outbreaks of greater proportions. The virus causes a potentially disabling illness known as Mayaro fever, which is similar to that caused by the chikungunya virus. The cocirculation of both viruses, with their clinical and structural similarities, and the absence of prophylactic and therapeutic measures highlight the need for studies that seek to understand the Mayaro virus. Using approaches *in silico*, we identified an antigenic and specific epitope (p_MAYV4) in domain A of the E2 glycoprotein of the Mayaro virus. This epitope was theoretically predicted to be stable and exposed on the surface of the protein, where it showed key properties that enable its interaction with neutralizing antibodies. These characteristics make it an interesting target for the development of immunodiagnostic platforms. Molecular dynamics simulation-based structural analysis showed that the PHE95 residue in the E1 fusion loop region is conserved among *Alphavirus* family members. PHE95 interacts with the hydrophobic residues of the E2 glycoprotein to form a cage-shaped structure that is critical to assemble and stabilize the E1/E2 heterodimer. These results provide important insights useful for the advancement of diagnostic platforms and the study of therapeutic alternatives.

## Introduction

The Mayaro virus (MAYV) is a neglected arbovirus belonging to the *Togaviridae* family and *Alphavirus* genus. It has a wildlife cycle that involves transmission between *Haemagogus* spp. mosquitoes and animal reservoirs^1,2^. MAYV is endemic to South America and has been reported in Central America^3,4^. Imported human cases have been detected in European and North American countries^2^. Climate and environmental changes may have contributed to its silent dispersion throughout Brazil and worldwide^1,4–7^. Detection of MAYV infections in dengue, Zika and chikungunya outbreaks, together with possible involvement of *Aedes* spp. in MAYV transmission, observed under laboratorial conditions, warns of the risk of outbreaks in naive populations^2,3,8–10^.

MAYV causes an acute and nonspecific febrile illness characterized by short viremia that can be accompanied by prodromal symptoms such as fever, headache, retro-orbital pain, vomiting, diarrhea, maculopapular rash, myalgia and arthralgia^2,11,12^. These symptoms are similar to those of other important arboviral diseases, such as chikungunya, dengue, Mayaro and Zika, suggesting a new term for this arboviral infection: “the ChikDenMaZika syndrome”^2^. More than 50% of patients develop debilitating and persistent joint pain during the chronic phase of the disease, similar to that caused by CHIKV infection^2^. Thus, developing sufficiently accurate diagnostic tests to distinguish infections caused by MAYV would be an important advance in regions where the arboviruses cocirculate.

The MAYV genome is composed of a positive-strand RNA approximately 11.5 kb in length and two open reading frames (ORFs). The first ORF is located in the genome 5′-end and encodes nonstructural viral proteins (nsP1, nsP2, nsP3, and nsP4) involved in viral replication and pathogenesis. The second ORF, positioned in the genome 3′-end, synthesizes the structural proteins of Capsid (C), envelope glycoproteins 1, 2 and 3 (E1/E2/E3) and a small 6 K protein, which are important for infection and protection of viral genetic material^13^. Structurally, the E1 and E2 glycoproteins have three domains interconnected by β-connectors. The E1 glycoprotein has 436 amino acids and three domains (I, II and III) distributed throughout the protein. Domain II is at the amino-terminal region, domain III is at the carboxy-terminal region and domain I is between domains II and III. The E2 glycoprotein has 422 amino acids and three domains (A, B and C). Domain B is positioned at the amino-terminal region of the protein; domain C is positioned at the carboxy-terminal region; and domain A is positioned between domains B and C^14–17^.

The E1/E2 glycoproteins are directly involved in the *Alphavirus* infectious process. The E2 glycoprotein recognizes and binds to a target receptor on the cell membrane to promote endocytosis^18–21^. The importance of the E2 glycoprotein was demonstrated by mutation studies in domain B of CHIKV and Semliki Forest virus (SFV)^19^. In *Alphavirus*, the acidic endosomal environment exposes and inserts the E1 fusion loop into the endosome membrane to trigger infection. This fusion results in viral capsid release into the cell cytoplasm, where it is disassembled and the viral genetic material is released, thereby inducing viral replication and polyprotein translation^15,18–20^. Cryogenic electron microscopy (cryo-EM) images of CHIKV^21,22^, Venezuelan Equine Encephalitis virus (VEEV)^23^, Barmah Forest virus (BFV)^24^ and Sindbis virus (SINV)^25^ show that E1/E2 glycoproteins form dimeric spicules on the membrane surface with regions exposed to the extracellular medium^24^. Exposure of E1/E2 glycoprotein residues to the extracellular environment enables their interaction with neutralizing antibodies, making them interesting targets for therapeutic and diagnostic studies^19,26–29^.

The development of a diagnostic platform for MAYV will be helpful in the clinical management of patients and especially for epidemiological screenings of a virus circulation pattern to prevent its spread^1^. The immediate obstacle in the development of this platform is the determination of a specific protein region that is adequate for making an accurate distinction among infectious agents^29^. However, studies have shown success by using small peptide sequences as antigens for diagnosing some *Flavivirus* and *Alphavirus*^29–32^. Bioinformatics and immunoinformatics tools can facilitate the rational search for these small peptide sequences within the viral proteome. Bioinformatics has been used in the rational design of drugs and vaccines to reduce the time and cost of their discovery and in the exploration of diagnostic platform designs for viral infections^33,34^.

To propose a peptide for application in MAYV immunodiagnostic tests, glycoprotein amino acid sequences from the MAYV and CHIKV E2 glycoproteins were analyzed according to their antigenic properties. Then, the three-dimensional structure of the E1/E2 heterodimer was predicted by molecular modeling and protein-protein docking strategies. Finally, the heterodimer produced was subsequently subjected to molecular dynamics (MD) simulations to analyze the heterodimer stability and understand the properties that could support a rationally designed peptide suitable for immunodiagnostic tests.

## Results

### MAYV E2 glycoprotein is highly conserved

The full amino acid sequences of MAYV and CHIKV E1 and E2 glycoproteins, isolated from different endemic countries, were retrieved from the Virus Pathogens Resource (ViPR) database (see Supplementary Table S1) and aligned in Mega 7.0 software. The alignment showed that the E1 and E2 glycoprotein sequences of MAYV strains are conserved and that there are important variations in some residues compared to the sequence of the CHIKV E2 glycoprotein. The greatest divergence in the MAYV sequences found in the three domains (3.3%) was obtained in a clinical sample acquired from Haiti in 2014. Representative sequences of MAYV (GenBank Access KM400591) and CHIKV (GenBank Access KP164567) were selected for antigenicity analyses and MD simulations.

### Peptide candidate as a potential antigen suitable for immunodiagnostic tests

Thirteen possible B-cell linear antigenic epitopes were identified in the E2 glycoprotein of MAYV and 11 possible epitopes were found in CHIKV using the Kolaskar and Tongaonkar antigenicity scale. The VaxiJen server identified seven antigenic regions of the MAYV glycoprotein and five antigenic regions of the CHIKV counterpart (Table 1 and see Supplementary Fig. S2).

**Table 1:** Predicted B-cell epitopes for E2 glycoproteins of Mayaro and Chikungunya viruses.

	Start	End	Peptide IEDB^a^	Length	VaxiJen Score^b^	
p_MAYV1	12	21	TRPYVAYCAD	12	−0.4159	MAYV
**p_MAYV2**	**88**	**94**	**SSECAVT** ^c^	**7**	**0.3806**	
p_MAYV3	100	105	FILAKC	6	−1.0822	
**p_MAYV4**	**107**	**116**	**PGEVISVSFV**	**10**	**0.7208**	
**p_MAYV5**	**148**	**156**	**GIELPCTTY** ^d^	**9**	**0.6357**	
**p_MAYV6**	**198**	**204**	**KYSCSCG** ^d^	**7**	**1.6669**	
p_MAYV7	222	231	VDKCQAYVTS	10	−0.1773	
p_MAYV8	254	259	VHIPFP	6	0.1979	
**p_MAYV9**	**266**	**274**	**RVPLAPEAL**	**9**	**0.7761**	
p_MAYV10	284	296	LSLHPIHPTLLSY	13	1.6888	
**p_MAYV11**	**317**	**325**	**TIPVPVEGV** ^**c**^	**9**	**−0.3628**	
p_MAYV12	355	361	IEYYYGL	7	0.5225	
p_MAYV13	365	393	TTIVVVVAVSVVVLLSVAASVYMCVVARN	29	0.5451	
p_CHIKV1	14	22	PYLAHCPDC	9	−0.1248	CHIKV
p_CHIKV2	27	35	SCHSPVALE	9	0.5933	
p_CHIKV3	47	54	KIQVSLQI	8	0.9054	
p_CHIKV4	82	88	GLFVRTS	7	1.0914	
p_CHIKV5	100	105	FILARC	6	−1.1297	
p_CHIKV6	122	128	SHSCTHP	7	0.2371	
p_CHIKV7	222	229	VDQCHAAV	8	−0.2062	
p_CHIKV8	225	269	VHIPFPLANVTCRVP	15	0.8076	
p_CHIKV9	284	295	IMLLYPDHPTLL	12	0.2245	
p_CHIKV10	354	362	EIILYYYEL	9	0.3660	
p_CHIKV11	367	389	TAVVLSVASFILLSMVGVAVGMC	23	0.7625	

^a^Peptides obtained from the online server Immune Epitope Database and Analysis Resource for the E2 glycoprotein from the Mayaro virus.

^b^Peptides obtained from the online server VaxiJen 2.0 for the E2 glycoprotein from the Mayaro virus.

^c^The online predictor VaxiJen 2.0 did not identify antigenicity for this peptide.

^d^Peptides have a sequence of residues conserved in the E2 glycoprotein of the chikungunya virus.

Among all antigenic regions predicted for MAYV, six peptides (p_MAYV2, p_MAYV4, p_MAYV5, p_MAYV6, p_MAYV9, p_MAYV11) were not predicted as antigenic regions in CHIKV. However, the peptide regions p_MAYV5 and p_MAYV6 may cross-react due to the presence of conserved residues in the CHIKV glycoprotein. The p_MAYV2 and p_MAYV11 peptides showed no antigenicity for MAYV according to the VaxiJen server. With the exception of the p_MAYV4 peptide, residues 107–116 in domain A, and p_MAYV9 peptide residues 266–274 in domain C, all the residues predicted for MAYV are conserved in the CHIKV E2 glycoprotein sequence (see Supplementary Fig. S3).

Thus, we consider that p_MAYV4 peptide (PGEVISVSFV) is a promising target for the development of a specific MAYV immunodiagnostic assay. To improve the antigenicity of the peptide, we hypothesized that the elongation of p_MAYV4 between residues 108–120 (GEVISVSFVDSKN), which showed an antigenicity score of 1.1595 according to VaxiJen. This peptide was named p_MAYV4a. The peptide was analyzed on the BLASTp online server (https://blast.ncbi.nlm.nih.gov/Blast.cgi?PAGE=Proteins), and no sequence overlap with any other *Alphavirus* was identified. Residue 107 (PRO) was not searched because its insertion in the sequence decreased the antigenicity score of the peptide (VaxiJen Score: 0.9877).

### Physicochemical properties of the p_MAYV4a peptide

The physicochemical properties of the p_MAYV4a peptide were predicted using the ProtParam tool (http://web.expasy.org/protparam/). According to this prediction analysis, the peptide is 1,33 kDa, has acidic features (pI 4.37) and is probably hydrophobic, even though the index was low (GRAVY score: 0.079). In addition, p_MAYV4a is possibly stable under natural conditions (the instability score was 6.92). The yeast half-life time *in vivo* exceeded 20 hours.

### Molecular docking of the E1/E2 glycoproteins of MAYV

Due to the absence of resolved MAYV E1/E2 glycoprotein structures, an initial three-dimensional (3D) model was produced for each protein using the I-TASSER server. The five major templates used for MAYV E1 glycoprotein modeling were VEEV (3J0C), SINV (3J0F), CHIKV (3N42), EEEV (6MUI), and BFV (2YEW), and for E2 glycoprotein, they were VEEV (3J0C), BFV (2YEW), SINV (3J0F), CHIKV (3N40) and CHIKV (2XFB) (see Supplementary Table S3).

The best models of the E1 and E2 glycoproteins showed C-scores of 2.0 and 1.91 and root mean square deviation (RMSD) values of 2.7 Å and 3.1 Å, respectively. The TM-score for both proteins was 0.99. The E1 glycoprotein monomer had 82.7% residues in favorable regions and 97.7% in allowed regions, while the E2 monomer had 81.9% in favorable regions and 96.0% in allowed regions. The values predicted by these analyses indicate that the models determined for the E1 and E2 glycoprotein monomers have a great chance of representing their expected native structures. Thus, from these monomers, it was possible to obtain the dimeric structure from molecular docking simulations performed by ClusPro 2.0. Among the ten structures produced by the ClusPro 2.0 server, we selected the one that presented the highest similarity with the resolved structures of the *Alphavirus* CHIKV^13^, VEEV^22^ and BFV^23^.

### Molecular dynamics analysis of the MAYV E1/E2 glycoprotein dimer

**Figure 1:**
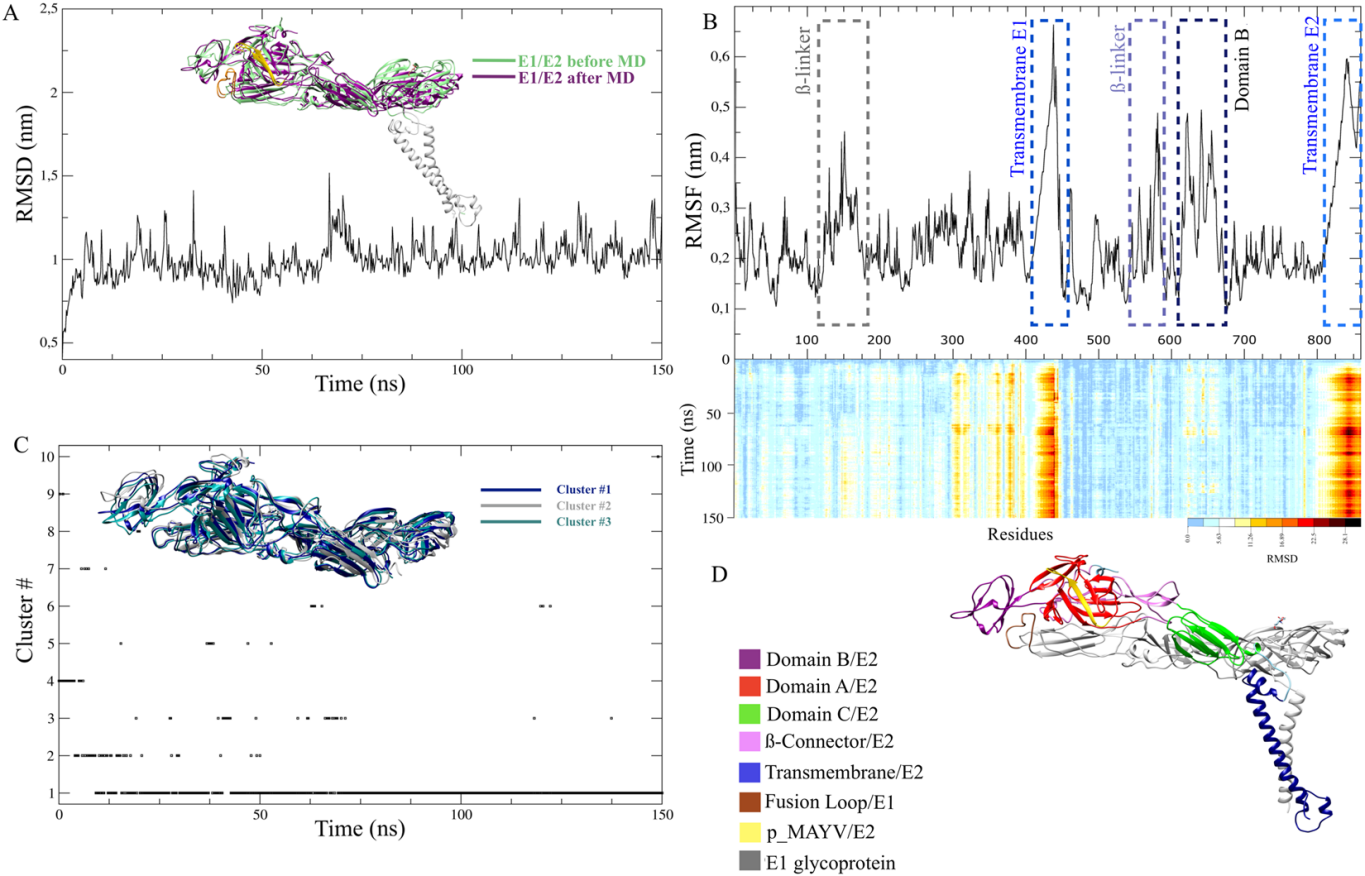
MD simulation of the MAYV E1/E2 glycoprotein heterodimer over 150 ns. (**A**) RMSD after MD simulations showing an increase in protein instability between ~ 60–75 ns and the subsequent stabilization of the heterodimer after this period. In the image, the E1/E2 dimer is shown before and after the MD simulation. Structural fluctuation is observed mainly in the E2 domain B. **(B)** Root mean square fluctuation (RMSF) plot showing the flexibility of the model of the E2 glycoprotein domain B (residues 610–675), a transmembrane region of the E1 glycoprotein (residues 400–442) and E2 glycoprotein (residues 800–859) and some β-linker regions. Below, the heatmap of RMSD value per residue as obtained during the 150 ns trajectory for the E1/E2 heterodimer. The scale shows the fluctuation of residues principally in the transmembrane regions of the monomers (darker colors). **(C)** Results from the cluster analyses of protein trajectories obtained during the simulation. A cut-off point of 0.25 nm was selected to include the major structures during the simulations. The graph shows the stabilization of cluster #1 after ~80 ns. The three main clusters (1, 2 and 3) obtained during the simulation overlap, highlighting fluctuations in the domain B region of the E2 glycoprotein. Note that the clusters oscillate mainly before the stabilization period of the RMSD, and after the stabilization period of the heterodimer (~80 ns), the predominance of cluster 1 is observed. **(D)** MAYV E1/E2 heterodimer model (cluster #1) in Ribbon, representing the most stable and frequent structure over the simulation. The E1 glycoprotein comprises residues 1–436 and the E2 glycoprotein residues 438–859. In the simulation, position 437 represents the glycan.

MD simulations of 150 nanoseconds (ns) for the predicted MAYV E1/E2 glycoprotein dimer and its behaviors were assessed by trajectory analysis. RMSD (root mean square deviation) was used to evaluate the deviation of the predicted models from the original states during the simulation (Fig. 1A). High-magnitude RMSD fluctuations throughout the simulation can be an indication of a flexible and mobile natural protein or the adjustment of the force field. According to the results of the analysis, Fig. 1A shows that the RMSD tends to stabilize at approximately 75 ns of the simulation, with approximately 1 nm deviating from the initial structure and a periodic fluctuation of low magnitude on the basis of this value.

A complementary measure of this parameter is performed by evaluating the fluctuations resulting from movements of each of the residues in a protein, which highlights the most flexible chain segments. Therefore, we evaluated the fluctuations of the residues in two ways: the mean fluctuation throughout the trajectory and that for the duration of each simulation. The root mean square fluctuation (RMSF) analysis of each residue was performed to determine which residues may have caused an increase in the RMSD values (see Fig. 1B). Significant fluctuations occurred in some loops of the β-linker region of the E1 glycoprotein (residues 120–175) and E2 glycoprotein (residues ~590), in the entire E2 B domain (residues 610–675) and, especially, in E1 (residues 400–442) and E2 (residues 800–859) transmembrane regions. In addition to the fluctuations determined by the RMSF analysis, a region of instability in E1 domain III (residues 300–390), near the membrane contact sites, was identified in an RMSD-per-residue heat map (Fig. 1B). Cluster analysis with a cut-off value of 0.25 nm showed the formation of eight different clusters (Fig. 1C), but after approximately 80 ns only the cluster number 1 was formed, which prevailed until the end of the simulation. Therefore, the central structure of cluster number 1 was defined in this work as the main structure of MAYV E1/E2, and it was similar to that observed in cryo-EM of other *Alphavirus*, such as CHIKV, VEEV and BFV.

The MAYV E1/E2 glycoproteins were formed mainly by conserved β-sheet and α-helix secondary structural components interspersed with small structures of bend, turn and coil in the regions that connected the E1 and E2 domains (see Supplementary Fig. S4A). Validation of the model generated by the MD simulation was performed by the MolProbity server, which presented results in Ramachandran plots (see Supplementary Fig. S4B). This structural analysis performed after the MD simulation revealed that 91.2% of the residues were in favorable regions and 98.9% were in allowed regions, showing refinement and improvement of the structure compared to the initial dimer model previously presented.

### The p_MAY4a peptide is stable and exposed at the surface of the MAYV glycoprotein dimeric structure

**Figure 2:**
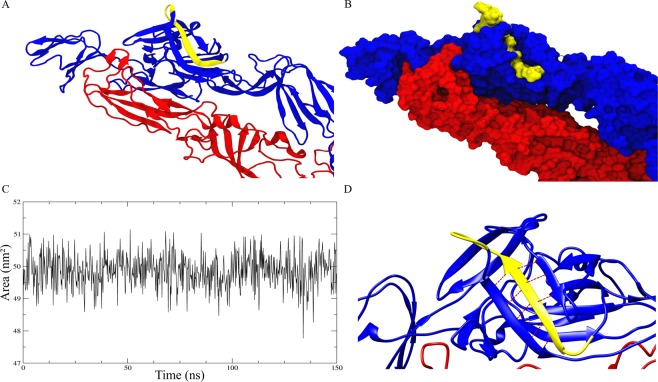
Analysis of the solvent-accessible surface area (SASA) of the p_MAYV4a peptide. (**A**) Ribbon structure showing the three-dimensional structure of the peptide in yellow in domain A of the E2 glycoprotein. It is noteworthy that the proposed peptide is composed of a β-sheet structure and a loop region responsible for increasing its antigenicity. **(B)** Visualization of the exposed peptide surface in the three-dimensional structure of the dimer. The p_MAYV4a peptide is yellow; E1 is red; and E2 is blue. The loop region forms a protrusion on the surface of the molecule into the surrounding environment, and the β-sheet region is buried inside the E2 glycoprotein. **(C)** Surface exposure of the peptide region (residues 545–557) along the simulation trajectory of 150 ns. The minimum area of exposure was 47.77 nm^2^, and the maximum was 51.4 nm^2^. **(D)** The Region of the peptide composed of hydrophobic residues (β-sheet) as seen in ribbon representation and showing hydrogen interactions (red dotted lines in the antiparallel structures).

The p_MAY4a peptide (PGEVISVSFVDSKN) is located in domain A of the E2 glycoprotein (108–120) (Fig. 1D). It is specific to MAYV and has a secondary structure organized as a β-sheet with short structural segments in bend and coil forms (see Supplementary Fig. S4A). The peptide has a protruding structure in the distal portion, which is exposed to the solvent and theoretically accessible to antibodies (Fig. 2A,B). The analysis of the solvent accessible exposure area (SASA) showed that the p_MAYV4a peptide exhibited solvent exposure that ranged from 47.77 to 51.14 nm^2^ throughout the simulation trajectory (Fig. 2C). The peptide has an aliphatic feature with two distinct regions: a β-sheet structure (residues 110–115), composed predominantly of hydrophobic side chain residues that establish hydrogen interactions with the neighboring antiparallel structure (residues 560–565, 521 and 523) (Fig. 2D), and a loop structure (residues 116–120) with hydrophilic side chain residues. Constant residue exposure and maintenance of the secondary structure of the peptide throughout the simulation were fundamental for the recognition of the neutralizing antibodies produced during MAYV infection.

### PHE95 and TRP89 residues in the E1 glycoprotein are important for MAYV E1/E2 dimer stabilization

**Figure 3:**
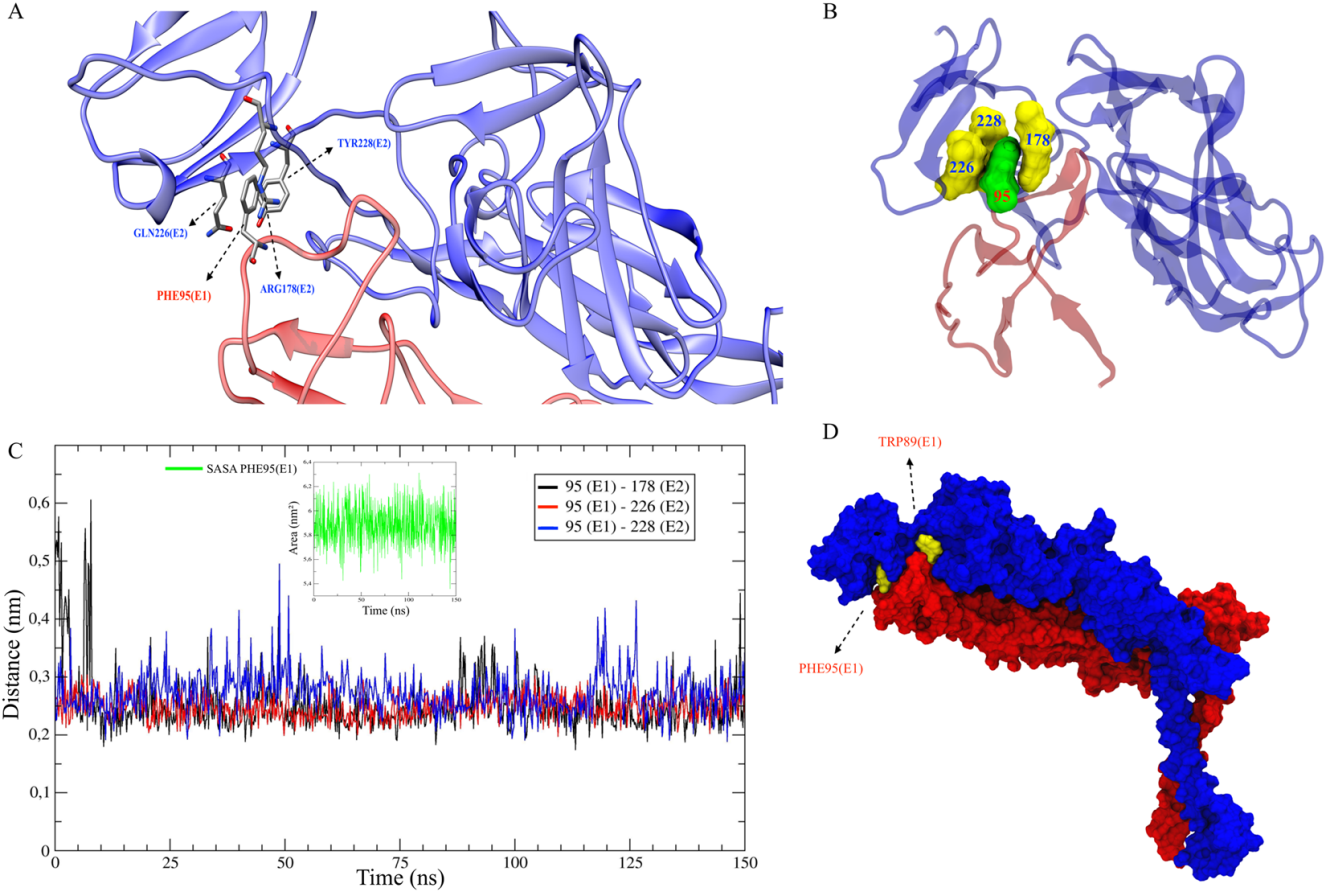
Region of contact between the E1 glycoprotein fusion loop and the E2 glycoprotein pocket. (**A)** Ribbon representation showing the formation of the cage-shaped structure by the residues GLN226, TYR228 and ARG178 of the E2 glycoprotein around the PHE95 residue in the E1 fusion loop. **(B)** Surface representation of residues GLN226, TYR228 and ARG178 of E2 (yellow) interacting with the PHE95 residue in the E1 fusion loop (green). The ribbon structure of the E1 glycoprotein is represented in blue, and the E2 glycoprotein is represented in red. **(C)** Plot showing the distance between residues ARG178, GLN226 and TYR228 relative to PHE95 in the fused region between the E1 and E2 monomers along the MD simulation trajectory of 150 ns. SASA of the PHE95 residue over the 150 ns simulation is shown in green (the SASA mean was 5.88 nm^2^). **(D)** Surface representation of the E1/E2 glycoprotein heterodimer highlighting residues PHE95 and TRP89 (yellow) that form a structure with clamp activity around the E2 glycoprotein (blue).

The PHE95 residue in the E1 fusion loop (domain I) is conserved among alphaviruses and interacts with the GLN226, TYR228 and ARG178 residues of E2 glycoprotein (domain B) in the binding crevice (Fig. 3A and Supplementary Video S1). These three residues flank and interact with the hydrophobic residue PHE95 (SASA mean of 5.88 nm^2^), retaining the fusion loop in the binding crevice between domains A and B (Fig. 3B,C). Finally, we identified the TRP89 residue in the fusion loop of the E1 glycoprotein, which is conserved among *Alphavirus* species and possibly involved in the formation of the dimer. TRP89 and PHE95 residues interact with the residues on the E2 glycoprotein during dimer formation. As shown in Fig. 3D, the PHE95 residue is buried in an E2 glycoprotein cavity, while the TRP89 residue is positioned externally (SASA mean of 23.70 nm^2^), thus forming a staple-like structure that fixes the E1 fusion loop to the E2 glycoprotein.

## Discussion

Arboviruses (ARthropod-BOrne VIRUS) are transmitted by arthropod vectors, such as mosquitoes and ticks^28^. These viruses are a challenge to public health authorities in endemic regions^6^. Continuous monitoring is required to avoid the dispersion of these viruses among naïve populations and prevent the emergence of new outbreaks^29^. MAYV is an emerging arbovirus in the Pan-Amazon region that causes restricted and self-limited outbreaks in South and Central America^2^. However, although *Haemagogus* spp. mosquitoes are the main vectors involved in the transmission of MAYV, *Aedes* spp. mosquitoes, found in peri-domiciliary areas of large cities in the Americas and Europe, may be involved in viral transmission. This possibility has raised concern with its dissemination as an important arboviral agent^1,2,6^.

The lack of accurate and simplified diagnostic platforms and prophylactic alternatives are obstacles to overcome^29,35–37^. The rational design of antigenic peptides using prediction tools *in silico* is a promising option in the development of immunodiagnostic platforms^29,32^. Therefore, this study was developed to fill two gaps related to MAYV knowledge: identify antigens and specific epitopes on the surface of the MAYV E2 glycoprotein for application in immunodiagnostic tests and provide a three-dimensional model of the E1/E2 glycoprotein dimer.

The results from the analyses of MAYV E2 glycoprotein *in silico* identified two potential antigenic linear peptides, the p_MAYV4 peptide present in domain A (residues 107–116) and the p_MAYV9 peptide inserted into domain C (residues 266–274). The p_MAYV4 peptide is more likely to be recognized by neutralizing antibodies produced during MAYV infection because domain A of the E2 glycoprotein has greater solvent exposure than domain C^17,35^. It is known that the neutralizing antibodies against *Alphavirus* are directed mainly to targets present in domains A and B of the E2 glycoprotein^21,26,28,35,36^. Therefore, due to the location of the p_MAYV4a peptide in domain A of the E2 glycoprotein, close to the cellular receptor binding site (THR58)^38,39^, we hypothesize that antibody binding in this region is associated with inhibited cell recognition.

The side chains of the hydrophobic amino acids in the β-sheet region (residues 110–115) of the peptide form hydrogen interactions with the neighboring antiparallel structure and provide stability to the peptide in the protein^40^. This interaction is important to ensure that the loop region of the peptide is continuously exposed to the solvent and to enable access by the antibody^41^. The loop region increased the antigenicity score of the p_MAYV4 peptide according to the VaxiJen server (score of 1.1595). This region is composed of four hydrophilic amino acid residues (ASP117, SER118, LYS119 and ASN120) arranged in a β-turn secondary structure at the amino-terminus. β-Turn secondary structures are more accessible to solvent and are more hydrophilic and flexible than β-sheets or α-helices. In addition, they have greater flexibility potential and hydrophilic scores, which are desired in the construction of an antigenic peptide^42,43^. The four residues were constantly exposed to solvent during the MD simulation. However, the insertion of the segment should be performed with caution since other *Alphaviruses*, such as CHIKV and VEEV, have a similar secondary structure^14,23,30,44^. Although these viruses have the same spatial arrangement in terms of the E2 glycoprotein structure, the primary sequence of the amino acid residues identified in CHIKV^30^ and VEEV is completely different from the one proposed for MAYV (p_MAYV4a).

The envelope proteins of Alphaviruses (E1/E2 glycoproteins) are fundamental during the infection process^17,20,25^; therefore, we proposed a structural model of the MAYV heterodimer generated by I-TASSER and submitted it for an MD simulation of 150 ns. The high protein RMSD value along the trajectory indicates that a significant change in the molecule, compared to the original structure, has occurred. However, the stability reached by the protein after 75 ns, persisting at a value of approximately 1 nm, illustrates its stability after the conformational adjustment. While RMSD is an analysis of the protein as a whole, the RMSF analysis describes the individual residue behavior along the trajectory. In this case, the RMSF analysis showed fluctuations in important regions of the molecule, which exemplifies the flexibility and mobility of the protein under study. The cluster analysis corroborates the RMSD profile when the stability indicated by the RMSD value is reached, and the time for the number of clusters to decrease is similar. It is noteworthy that, after 75 ns, only three clusters appear, and cluster number 1 is the most prevalent among them. This shows that cluster number 1 represents the most stable protein structures. In view of this finding, the central structure is determined to be cluster number 1, which is defined as the structure with the smallest average RMSD compared to all other structures of the cluster and resulted in a protein similar to the crystal structures of other species of *Alphavirus*^13,22–24^; thus, it was used as the MAYV E1/E2 model for this study.

Structurally, domain B is located at the distal end in relation to the membrane, positioned above domain II of E1 and protecting the fusion loop at neutral pH. The fluctuation observed in this domain may be due to a natural flexibility caused by its long β-linker^15^ or by the absence of glycoprotein E3, positioned next to domains A and B. When E3 glycoprotein is present, domain B remains stable and protects the fusion loop, but after cleavage, this domain moves to the membrane fusion^14,22^. The absence of the trimeric structure in the presence of neutral conditions (pH 7.0) during the simulation may have triggered instability in domain B and in the fusion loop. To evaluate this possibility, further studies of MD with the MAYV heterotrimer (E1, E2 and E3) will be carried out in different pH conditions to accurately determine the position and behavior of domain B in the viral particle throughout the infectious stages.

Cryo-EM studies show that the flexibility of domain B in *Alphavirus*, such as CHIKV, Sindbis virus and SFV, is necessary for them to bind to cellular receptors during the infectious process. In this scenario, domain B and its β-connectors move to expose the E1 fusion loop. The high values of RMSD and RMSF obtained in this study show equal flexibility of domain B of the MAYV. Our results also indicate that the movement observed in the regions of β-connectors may be useful for the protein to adapt to the environment during the fluctuations of domain B until a moment of stability is found.

Since there are no vaccines available that neutralize the infection by *Alphavirus*, the movement described in domain B of the MAYV is an interesting target to be explored for the development of vaccines using neutralizing antibodies. Neutralizing antibodies produced against the epitope described in this work or that recognize other regions of domain B, domain A, or the β-connectors may be sufficient to prevent their movement, exposure of the E1 fusion loop and consequent viral infection. Currently, some works explore the development of antibodies against Alphavirus and describe that antibodies produced against domain B, domain A or β-linkers are highly effective in neutralizing the infection^16,21,25,35,36^. However, these works do not exploit peptide regions of the glycoproteins for the determination of targets for neutralizing antibodies. In this study, we explored an approach *in silico* that enabled us to characterize the E1/E2 glycoprotein structures, to identify important movements and behaviors of the virus, and to identify an antigenic region in domain A.

A previous CHIKV study showed that residues HIS29, HIS73 and HIS226 of the E2 glycoprotein contribute to dimer stability^14^. Two of those residues (HIS29 and HIS73) are conserved in MAYV, but not HIS226, substituted by H226Q. Our analysis adds that the amino acid PHE95, present in the E1 fusion loop, is conserved among *Alphavirus* and is important for dimer fixation when interacting with the amino acids TYR228, ARG178 and GLN226 that are present in domain B and in the β-linker of the E2 glycoprotein. The MD simulation results show that these three residues form a cage-like structure that theoretically imprisons the nonpolar residue PHE95. This result will be evaluated by alanine scanning and experiments *in vitro* in the future. If the peptide predicted in this work is confirmed, then PHE95 is a promising therapeutic target candidate.

In summary, our results highlight an antigenic sequence in the MAYV E2 glycoprotein with potential for the development of immunodiagnostic platforms. In addition, they suggest a structural model of the MAYV E1/E2 heterodimer with insights for a better understanding of its structure and behavior, making it a hot topic for further studies involving mutagenesis and drug therapy against MAYV and CHIKV, major arthritogenic *Alphaviruses*. Finally, we emphasize that these results will be applied in subsequent confirmatory *in vitro* tests.

## Methodology

### Sequences of the E1 and E2 glycoproteins of MAYV and CHIKV

The methodological flowchart is shown in Supplementary Fig. S5. Complete E1 and E2 glycoprotein amino acid sequences of MAYV and CHIKV were obtained from the Virus Pathogens Research (ViPR) database (https://www.viprbrc.org) and aligned using Muscle software implemented in the Mega 7.0 program^45^. Representative sequences of MAYV (GenBank KM400591.1) and CHIKV (GenBank KP164567) were selected for further analysis.

### Prediction of continuous linear B-cell epitopes

MAYV linear B-cell epitopes were predicted using the Kolaskar and Tongaonkar antigenicity scale^46^ (http://tools.immuneepitope.org/bcell/). The Kolaskar and Tongaonkar antigenicity scale is a semiempirical epitope prediction method with more than 75% prediction accuracy^46^. Peptides that reached or crossed the threshold of 1.05 were classified as potential antigenic epitopes^47^. Potential antigenic sequences specific for MAYV as determined through the use of the antigenicity scale of Kolaskar and Tongaonkar were submitted to a second online antigenicity prediction platform, VaxiJen (http://www.ddg-pharmfac.net/vaxijen)^48^. The VaxiJen server is an alignment-independent antigen predictor with 87% viral epitope prediction accuracy^47^. These antigen prediction methodologies are based on the physicochemical properties of amino acid residues and their frequency of identification in previous experimental studies^45,46^.

### Prediction of the physicochemical properties of epitopes

Physicochemical properties of the MAYV antigenic sequences, including half-life, instability index, aliphatic index, theoretical pI and the hydropathicity value, were predicted using the ProtParam online tool^49^ (http://web.expasy.org/protparam/). The half-life prediction estimates how long a peptide remains stable in prokaryotic and eukaryotic organisms. A protein is considered stable-when the value obtained is lower than the cut-off value of 40, while the hydropathicity index evaluates the probability of a region being hydrophobic (positive values) or hydrophilic (negative values)^32^. The secondary structure of the peptide was assessed by a graphic representation generated by the MD.

### Prediction of the 3D structure of the Mayaro virus E1 and E2 monomers and glycoprotein dimer docking

Due to the absence of resolved dimer and monomer structures of the MAYV E1/E2 glycoprotein in the PDB (Protein Data Bank), a three-dimensional (3D) structural model was generated using a threading modeling methodology on the I-TASSER online prediction server^50^ (https://zhanglab.ccmb.med.umich.edu/I-TASSER/). Based on the primary amino acid sequences of select E1 and E2 glycoproteins (KM400591.1), the I-TASSER determines the 3D structure by iterative simulations of segmentation assembly^50–52^.

The quality of the structural model generated by I-TASSER was evaluated with the MolProbity server^53^ (http://molprobity.biochem.duke.edu/index.php). This server uses two measures to evaluate the quality of the produced model: the clashscore, which evaluates the number of severe steric overlays per 1,000 atoms, and the MolProbity value, which combines the clashscore, rotameter and Ramachandran ratings into a single score that is normalized such that it is on the same scale as the X-ray resolution. These combined measures enable the quality assessment of a geometric model^53^.

For the formation of the E1/E2 dimeric structure of the MAYV envelope glycoprotein, the best models produced by the I-TASSER were submitted to the ClusPro 2.0 online server^54^ (https://cluspro.bu.edu/login.php). The server performs molecular docking according to free energy parameters and produces 10 models ordered according to the structure requiring the lowest free energy for docking. The best output hits from ClusPro were submitted for refinement and analysis of the MD simulation^53^.

### Molecular dynamics simulations of the dimeric E1/E2 glycoprotein of the Mayaro virus

Correct disulfide bonds between cysteine residues in the I-TASSER 3D model were determined by the tleap tool using the AMBER ff14SB force field from the AMBER18 package^55,56^. The sugar residues were fixed using the GLYCAM_06j-1force field^57^ (http://glycam.org/) and histidine residues predicted by the H++ server^58^ (http://biophysics.cs.vt.edu/credits.php) were protonated using the AmberTools^55^. The protein format was converted using ACPYPE^59^, and the system was minimized and equilibrated with a TIP3P water model and CL^−^ ions^60^. Transmembrane protein domains at residues 397–436 in the E1 glycoprotein and 346–422 in E2 glycoprotein were restricted throughout the simulation. The MD simulation was performed using GROMACS 5.1.2 software^61^ in the AMBER ff99SB-ILDN force field^62^. The system was subjected to 150 ns simulation at 300 k temperature and 1 bar pressure, with restriction confirmed for only the transmembrane domain. The trajectory analysis was performed using RMSD and RMSF, calculated from GROMACS tools package, in the gromos algorithm. The RMSD is a measure of the spatial difference between two static structures, and in the simulation, the calculation was performed on the basis of the initial structure and all succeeding trajectory frames. On the other hand, the RMSF profile calculates the flexibility of a residue based on the fluctuation around an average position among all MD simulations^63^. The g_cluster (GROMACS) program, based on the RMSD profile with a cut-off of 0.25 nm, was used to determine the conformations that were found most frequently along the trajectory. In this case, all structures with RMSD values less than 0.25 nm for any element in a cluster are added to the primary cluster. It is unlikely that a molecule with an RMSD value higher than 0.25 nm from another cluster would be considered a structure. UCSF Chimera^64^ and Visual Molecular Dynamics (VMD)^65^ were used to visualize protein behavior throughout the simulation. The quality of the proposed model was evaluated using the MolProbity server^51^.

## Supplementary information

Video 1

Supplementary Information
