# Supplementary material for: Prediction of MAYV peptide antigens for immunodiagnostic tests by immunoinformatics and molecular dynamics simulations
Source: Sci Rep. 2019 Sep 16;9:13339. doi: 10.1038/s41598-019-50008-3 (PMC6746749; doi:10.1038/s41598-019-50008-3)
Supplement: Supplementary file 2 — Supplementary Information [file 41598_2019_50008_MOESM2_ESM.doc]

**Prediction of MAYV** **peptide antigens for immunodiagnostic tests by immunoinformatics and Molecular Dynamics simulations**

RODRIGUES, Roger Luiz^1^; MENEZES, Gabriela de Lima^2^; SAIVISH, Marielena Vogel^1^; COSTA, Vivaldo Gomes da^3^; PEREIRA, Maristela^4^; MORELI, Marcos Lázaro^1*^ and SILVA, Roosevelt Alves da^2*^.

^1^Universidade Federal de Goiás, Laboratório de Virologia, Jataí, GO, 75801-615, Brazil.

^2^Universidade Federal de Goiás, Núcleo Colaborativo de BioSistemas, Jataí, GO, 75801-615, Brazil.

^3^Universidade de Brasília, Departamento de Biologia Celular, Brasília, DF, 70910-900, Brazil

^4^ Universidade Federal de Goiás, Laboratório de Biologia Molecular, Instituto de Ciências Biológicas, Goiânia, GO, 74690-900, Brazil.

***Correspondent authors:** Roosevelt Alves da Silva (rooseveltfisicaufg@gmail.com) and Marcos L. Moreli (marcos_moreli@ufg.br)

**Supplementary Table S1: E1 and E2 protein sequences of Mayaro and Chikungunya viruses from human samples.**

| **Country** | **Year** | **GB access** | **Virus** |
| --- | --- | --- | --- |
| Brazil | 2004 | KM400591 | MAYV_E2 |
| Trinidad and Tobago | 1954 | MK070492 |
| French Guiana | 2013 | KJ013266 |
| Haiti | 2014 | KY985361 |
| Peru | - | KY026196 |
|  |  |  |  |
| Bangladesh | 2014 | MF773566 | CHIKV_E2 |
| Nicaragua | 2015 | KY703978 |
| USA | 2014 | KY680368 |
| Italy | 2017 | MG049915 |
| India | 2011 | KJ679577 |
| Brazil | 2014 | KP164567 |
| Micronesia | 2013 | KJ451623 |
| Dominican Republic | 2014 | KY272964 |
| Philippines | 2014 | MF773563 |
|  |  |  |  |
| Brazil | 2015 | MH5135597 | MAYV_E1 |
| Trinidad and Tobago | 1954 | MK070492 |
| French Guiana | 2013 | KJ013266 |
| Haiti | 2014 | KY985361 |
| Peru | - | KY026195 |
|  |  |  |  |
| Bangladesh | 2018 | FJ807898 | CHIKV_E1 |
| Nicaragua | 2015 | KY703891 |
| USA | 2014 | KY680384 |
| India | 2016 | KY057363 |
| Brazil | 2014 | KP164568 |
| Dominican Republic | 2014 | KY272966 |
| Philippines | 2013 | AB860301 |
| Indonesia | 2015 | KX097982 |
| New Caledonia | 2011 | HE806461 |

**Supplementary Table S2:** Amino acid punctual substitutions in the Mayaro Virus E2 glycoprotein.

| **Country/Year** | **GenBank access** | **Amino acid substitutions** | **Domains** |
| --- | --- | --- | --- |
| Brazil/2004 | KM400591 | V197I  T216I | B |
|  |  |  |  |
| Trinidad and Tobago/1954 | MK070492 | T59K  H130R | A |
|  |  | I175V  T196I | B |
|  |  |  |  |
| French Guiana/2013 | KJ013266 | S374T | T |
|  |  |  |  |
| Peru/- | KY026196 | T365A | T |
|  |  |  |  |
| Haiti/2014 | KY985361 | V37I  D74Y  A92T  V110A  Q122H  Y129F | A |
|  |  | K198R | B |
|  |  | R301A  T312A  R328Q | C |
|  |  | R346K  T365I  V371I  V381L | T |

**Supplementary Table S3: Protein structure quality scores and threading templates information.**

| **TEMPLATES** |  | **GLYCOPROTEIN** | | | | | | | | |
| --- | --- | --- | --- | --- | --- | --- | --- | --- | --- | --- |
|  |  | **E1** | | | |  | **E2** | | | |
|  |  | **TM-score** | **RMSD** | **IDEN^*^** | **CONV^#^** |  | **TM-score** | **RMSD** | **IDEN^*^** | **CONV^#^** |
| **3J0C** |  | 0.98 | 1.12 | 0.52 | 1.00 |  | 0.97 | 1.28 | 0.37 | 0.99 |
| **3J0F** |  | 0.91 | 2.36 | 0.45 | 0.98 |  | 0.82 | 3.44 | 0.32 | 0.95 |
| **3N42** |  | 0.85 | 1.82 | 0.62 | 0.90 |  | - | - | - | - |
| **6MUI** |  | 0.83 | 2.36 | 0.58 | 0.91 |  | - | - | - | - |
| **2YWE** |  | 0.80 | 3.39 | 0.44 | 0.94 |  | 0.83 | 3.54 | 0.38 | 0.96 |
| **3N40** |  | - | - | - | - |  | 0.76 | 2.08 | 0.56 | 0.81 |
| **2XFB** |  | - | - | - | - |  | 0.69 | 2.65 | 0.48 | 0.76 |

*Identity is the percentage sequence identity of the whole template chains with query sequence. ^#^Coverage represents the coverage of the threading alignment and is equal to the number of aligned residues divided by the length of query protein.

**
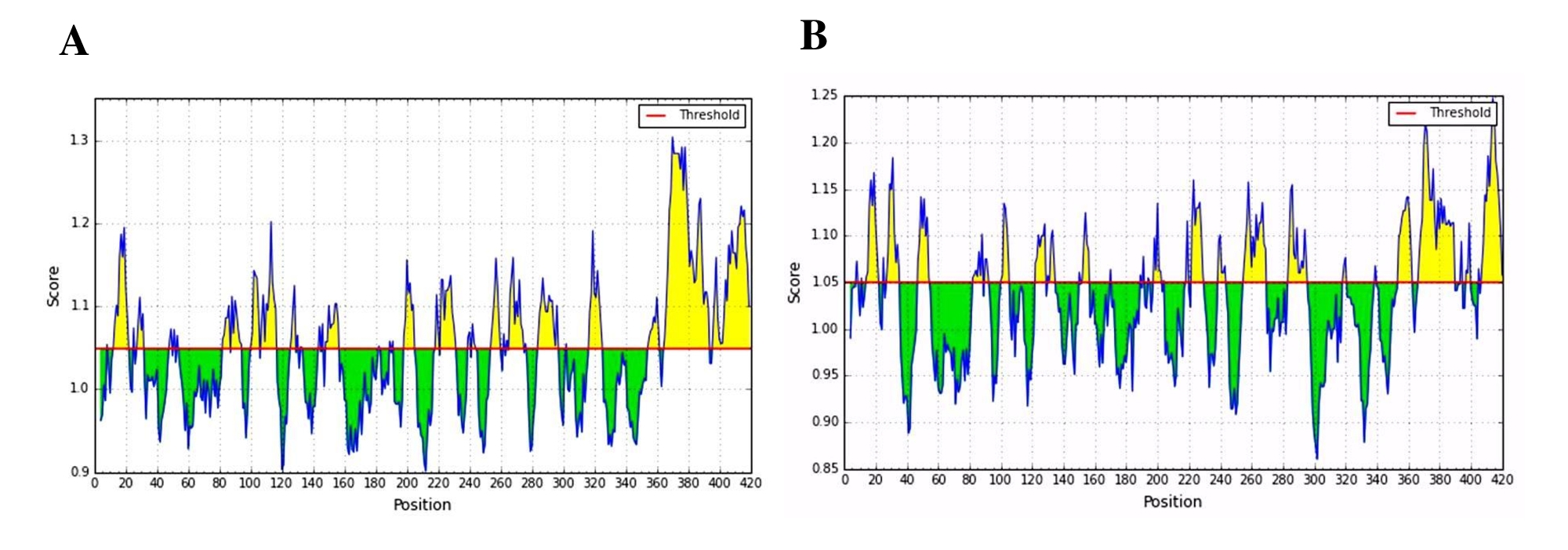
**

**Supplementary Figure S2: Antigenicity prediction of MAYV (A) and CHIKV (B) E2 glycoproteins by the Immune Epitope Database (IEDB).** In the horizontal axis is presented the position of each amino acid in the sequence and in the vertical axis the probability of certain region in the glycoprotein to be antigenic (Threshold = 1.05). The yellow and green bars show regions of probable antigenic and non-antigenic sequences, respectively.


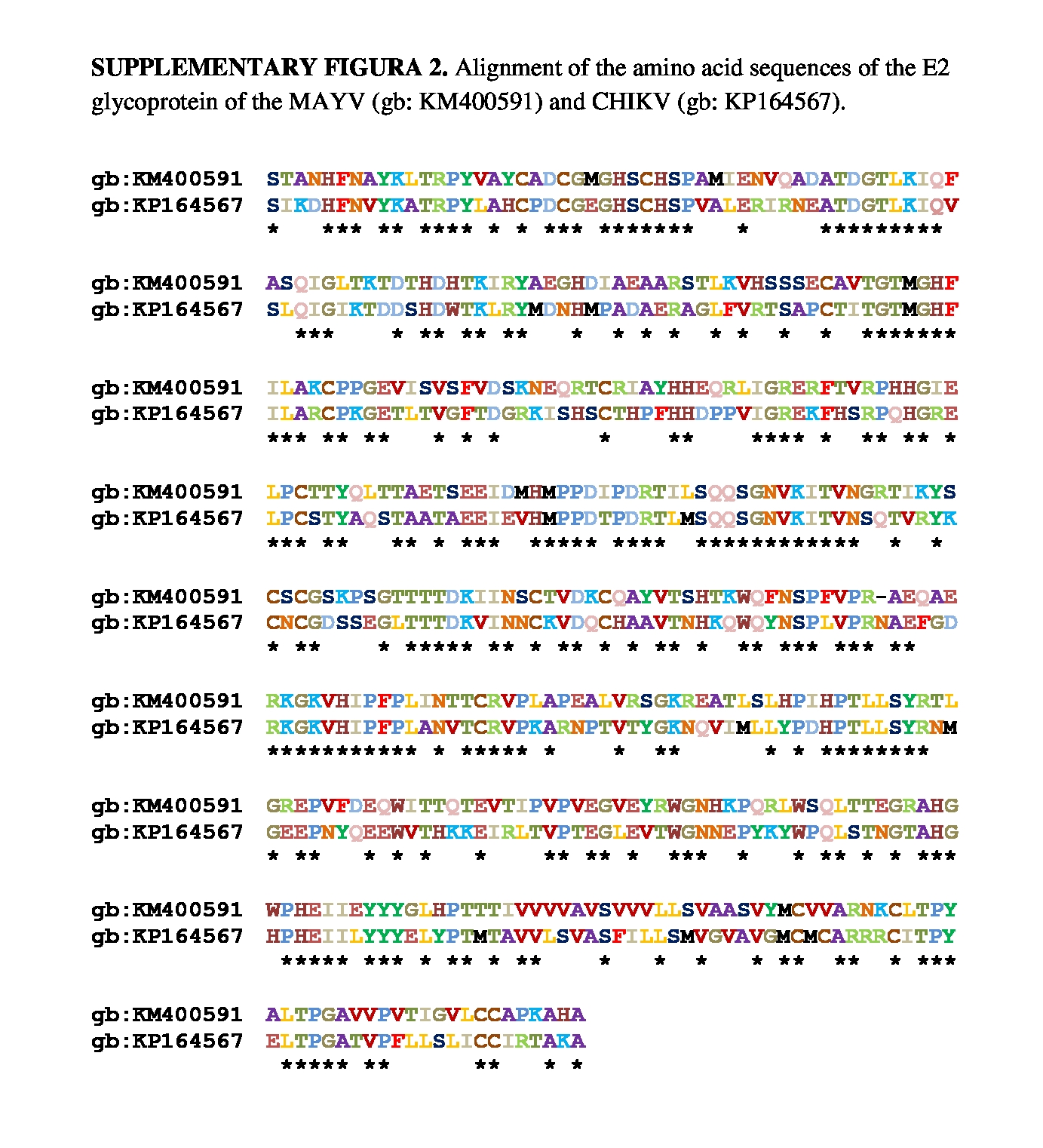


**Supplementary Figure S3. Alignment of the sequence of the MAYV (gb:KM400591) and CHIKV (gb: KP164567) used for antigenicity and Molecular Dynamics simulation analyzes.** The sequence is distributed on the image so that each line contains 50 amino acid residues. The peptide p_MAYV4a is composed of the residues 108-120 and highlighted by red dashed lines.


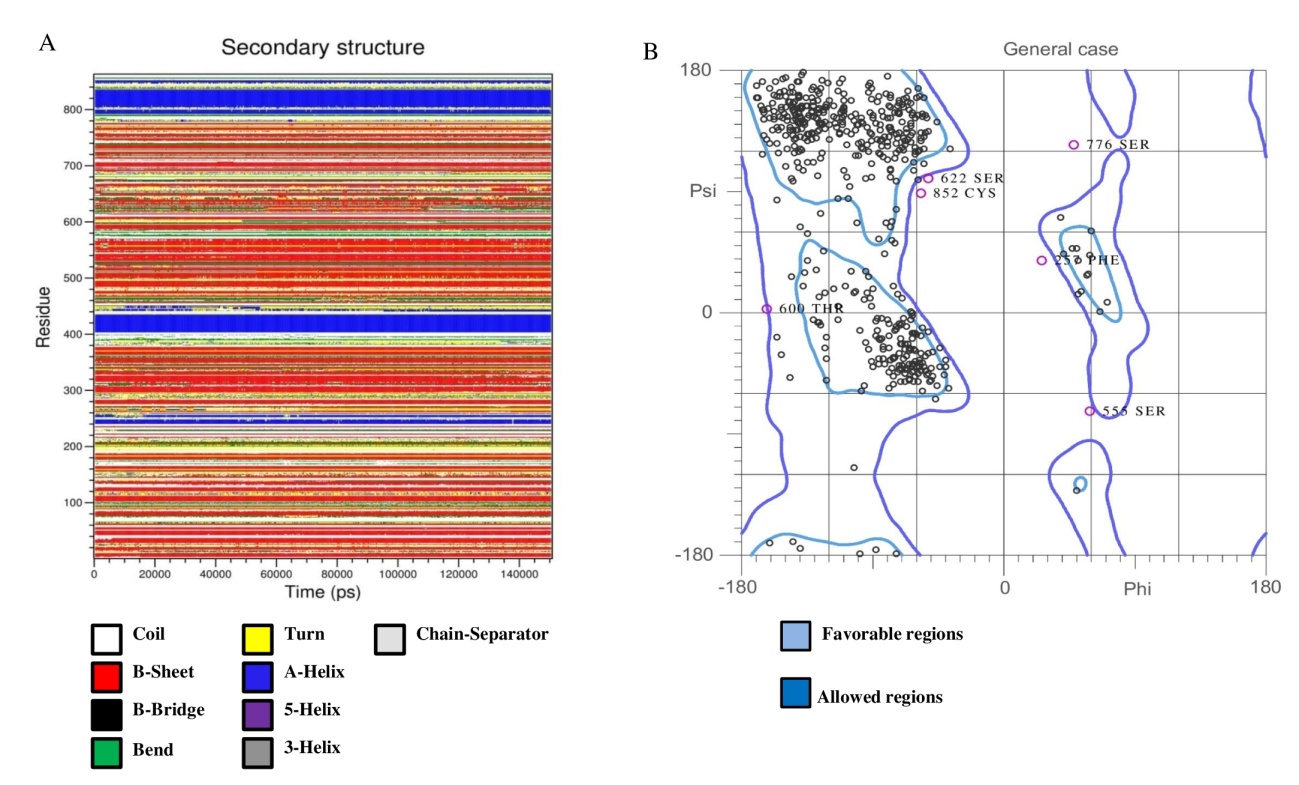


**Supplementary Figure S4. Evaluation of the dimeric structure of the MAYV E1/E2 glycoprotein model. A)** Secondary structure of the MAYV E1/E2 glycoprotein heterodimer showing highly conserved secondary structures throughout the simulation of 150 ns. **B)** Validation of the tertiary structure of the MAYV E1/E2 glycoprotein heterodimer by Ramachandran plot showing that 91.2% of the residues are in favored region, 98.9% of residues in allowed region.

**Supplementary Video S1: Behavior of PHE95 residue (E1) throughout the simulation**. In blue is highlighted the E2 glycoprotein with its A and B domain and in red the E1 fusion loop positioned between the domains of the E2 glycoprotein. Observe the movement of the aromatic amino acid PHE95 in the fusion loop interacting with the hydrophobic residues GLN226, TYR228 and ARG78 of E2, forming a trapping structure for PHE 95.


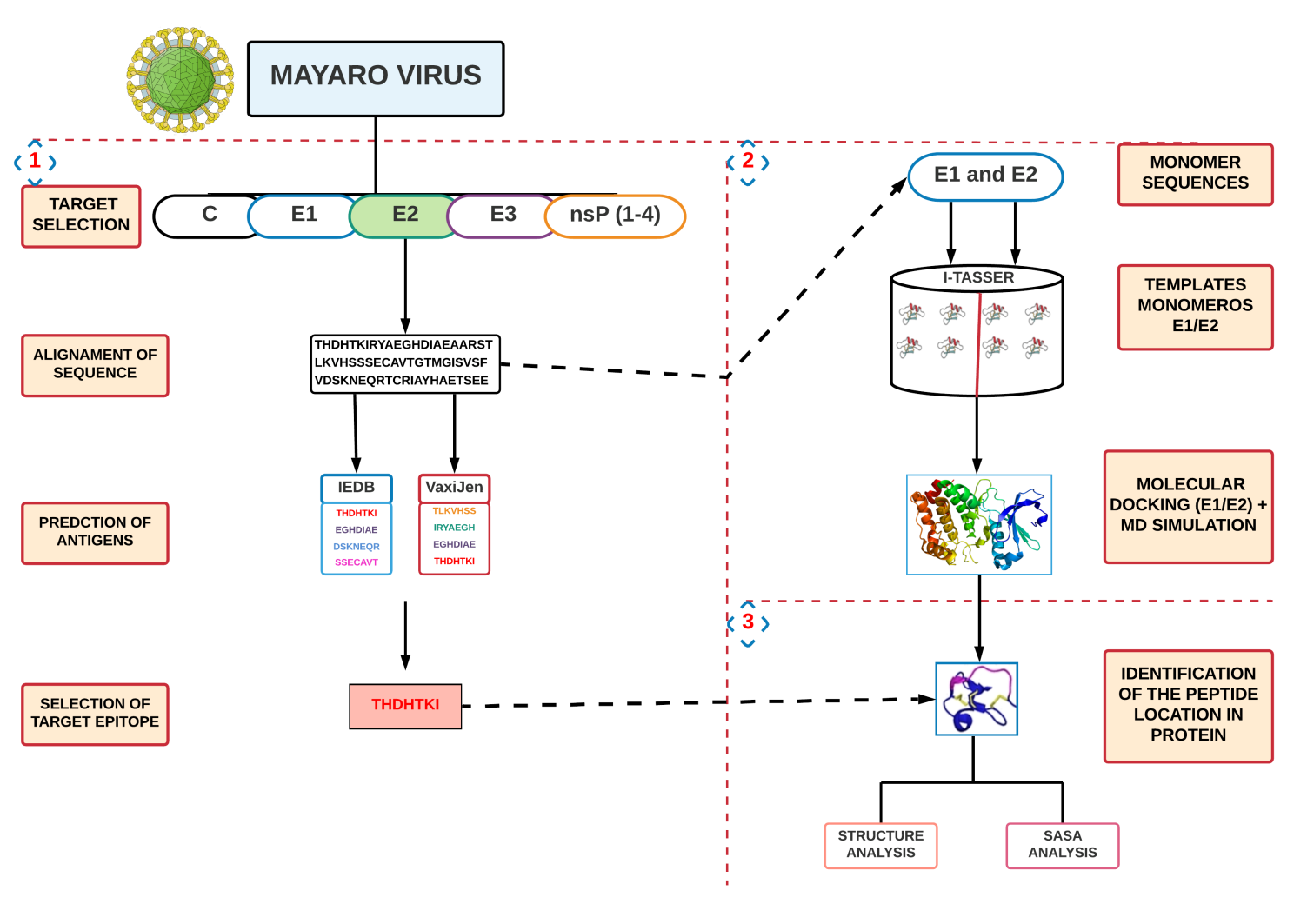


**Supplementary Figure S5:** Flowchart of the prediction of antigenic peptides using in silico approach. The protocol consists of three modules: (1) Module 1 consists of the steps of determination of the target protein, alignment of the target protein amino acid sequences deposited in the VipR database, submission to the IEDB and VaxiJen antigen prediction servers; 2) Module 2 consists of the protein modeling steps using the I-Tasser server, molecular docking by the ClusPro server and MD simulation; (3) The third module is the analysis of the position of the target peptide in the resolved protein and analysis of the structure and the Solvent Exposure Area of the selected peptide.
